# An Efficient Antipodal Cell Isolation Method for Screening of Cell Type-Specific Genes in *Arabidopsis thaliana*

**DOI:** 10.1371/journal.pone.0166390

**Published:** 2016-11-22

**Authors:** Xiaobo Yu, An Luo, Meng-xiang Sun

**Affiliations:** 1 Department of Cell and Developmental Biology, College of Life Science, State Key Laboratory of Hybrid Rice, Wuhan University, Wuhan, 430072, China; 2 College of Life Science, Yangtze University, Jingzhou, 434023, China; Beijing Forestry University, CHINA

## Abstract

In flowering plants, the mature embryo sac consists of seven cells, namely two synergid cells and an egg cell at the micropylar end, one central cell, and three antipodal cells at the chalazal end. Excluding the antipodal cell, as a model for the study of cell fate determination and cell type specification, the roles of these embryo sac component cells in fertilization and seed formation have been widely investigated. At this time, little is known regarding the function of antipodal cells and their cell type-specific gene expression patterns. One reason for this is difficulties related to the observation and isolation of cells for detailed functional analyses. Here, we report a method for antipodal cell isolation and transcriptome analysis. We identified antipodal cell-specific marker line K44-1, and based on this marker line, established a procedure allowing us to isolate antipodal cells with both high quality and quantity. PCR validation of antipodal-specific genes from antipodal cell cDNA showed that the isolated cells are qualified and can be used for transcriptome analysis and screening of cell type-specific marker genes. The isolated cells could keep viable for a week in culture condition. This method can be used to efficiently isolate antipodal cells of high quality and will promote the functional investigation of antipodal cells in *Arabidopsis thaliana*. This increases our understanding of the molecular regulatory mechanism of antipodal cell specification.

## Introduction

In flowering plants, the female germ lineage initiates from a hypodermal cell located at the tip of the ovule primordium [1, 2]. This cell quickly differentiates into a megaspore mother cell. The megaspore mother cell undergoes meiosis and gives rise to four haploid megaspores. Three megaspores undergo programmed cell death and the remaining one forms the functional megaspore, which further undergoes three rounds of nuclear division and becomes a large eight-nucleate coenocytic cell [3, 4]. After cellularization, this large coenocytic cell develops into a mature embryo sac with seven cells of four cell types: two synergid cells and one egg cell at the micropylar pole, one central cell with two nuclei, and three antipodal cells at the chalazal pole [2].

Since the embryo sac is located where fertilization and embryogenesis occur, the roles of its component cells in fertilization and seed development have been studied for more than a century. However, of all four cell types in the mature embryo sac, antipodal cells are rarely investigated. It was proposed that antipodal cells are dispensable for fertilization and seed development in *Arabidopsis thaliana* [2, 4]. However, it was reported that the antipodal cells are not really degenerated right after fertilization as we previously thought [5]. It was also reported that ZmEAL1, secreted by egg cells, influences antipodal cell fate [6], indicative of a relationship between antipodal cells and other cells in the embryo sac (they are not just a bystander). In lachesis (LIS) mutants, the cell fate of egg and synergid cells changes and three antipodal cells enlarge and fuse to form a central cell-like cell [7], suggestive of potential for antipodal cell fate transition. In rice and some grass species the antipodal cells were found quite active, they proliferate quickly and even undergo endoreduplication [8]. These studies suggest that antipodal cells are found in the embryo sac and during seed development; thus, it is important to explore their role in these processes.

Although some antipodal cell-specific genes have been isolated via differential expression screens of wild-type ovules and determinant infertile (dif1) ovules [9], little is known regarding the transcript landscape of antipodal cells. Meanwhile, as antipodal cells are embedded deeply in the sporophytic tissues and located close to the chalazal pole of an embryo sac, it is difficult to follow their developmental process and isolate them from ovules for detailed analyses, which is a major obstacle for the application of modern research techniques. Therefore, it is important to establish a proper technique to overcome this obstacle.

Here, we report an efficient procedure to isolate antipodal cells in *Arabidopsis thaliana*. This method allows us to isolate a population of antipodal cells from living ovules without fixation, which facilitates observation, *in vitro* culture, and transcriptome analysis.

## Materials and Methods

### Materials

Arabidopsis plants were grown on soil in a greenhouse under long-day conditions (16 h light/8 h dark) at 22°C.

#### Vector construction and plant transformation

The *DD13* promoter was PCR-amplified from genomic DNA and cloned into PART27 backbone-containing EGFP and H2B element. The vector was introduced into *Agrobacterium* strain GV3101 by electroporation. *Arabidopsis* plants (ecotype Columbia) were transformed using the floral dip procedure [10].

#### Preparation for antipodal cell isolation

The flower stage is determined according to previous work [11]. Prior to antipodal cell isolation, enzyme buffer, razor blades, glass capillaries, a Petri dish (Φ 3.5 cm), and 2x lysis buffer should be prepared for use, as described previously [12]. Enzyme buffer contains 10.5% (w/v) mannitol with 1% (w/v) cellulose (Yakult Honsha Co. Ltd, Tokyo, Japan) and 0.8% (w/v) Macerozyme (Yakult Honsha), pH 5.8. The washing buffer is 10.5% (w/v) mannitol. The 2x lysis buffer is a mixture of 200 mM Tris-HCl, pH 7.5, 1 M LiCl, 20 mM EDTA, pH 8.0, 2% LiDS, and 10 mM dithiothreitol (DTT).

#### EGFP imaging and microscopy for isolation

The ovules and isolated antipodal cells were analyzed using a FV1000 confocal laser-scanning microscope (CLSM; Olympus). The isolation process was executed under an Olympus IX71 fluorescent microscope.

#### mRNA extraction and cDNA amplification

mRNA of antipodal cells was extracted using Dynabeads mRNA DIRECT Micro Kit (Invitrogen) according to the manufacturer’s instructions. Reverse transcription and cDNA amplification were executed using the SMARTer Ultra Low Input RNA Kit for Sequencing—v3 (Clonetech) per the manufacturer’s protocols.

#### Cell culture

The antipodal cells were isolated as described above. The isolated antipodal cells were first put into a droplet of isolation buffer and observed by confocal laser-scanning microscope (CLSM, Leica) to record the basic character of the cell before culture. Then the antipodal cells were cultured in Km8p medium according to previously established method [13] with some modifications. In detail, the microchamber (MILLCELL-CM, MILLPORE, 0.4μm) was first put into a 3cm Petri dish containing 1.5mL culture medium A (MS + Km8p-Vitamin +0.3M sucrose+0.05M mannitol +0.05M sorbitol +3mM MES), then 100μL culture medium B (MS + Km8p-Vitamin +0.3M sucrose+0.1M mannitol +0.1M sorbitol +3mM MES + 0.5mg/L NAA + 0.25mg/L 6-BA) was added into the microchamber. The isolated antipodal cells after washing in sterile isolation buffer were transferred into the microchamber for culture. Around 100 ovules at the stage just after fertilization were put into the Petri dish as the feeder cells. The antipodal cells were cultured in the dark at 25°C. The cultured cells were then observed by IX71 microscope (Olympus) and confocal laser-scanning microscope (CLSM, SP8, Leica)

## Results

### Establishment of Antipodal Cell Marker Line

**Fig 1:**
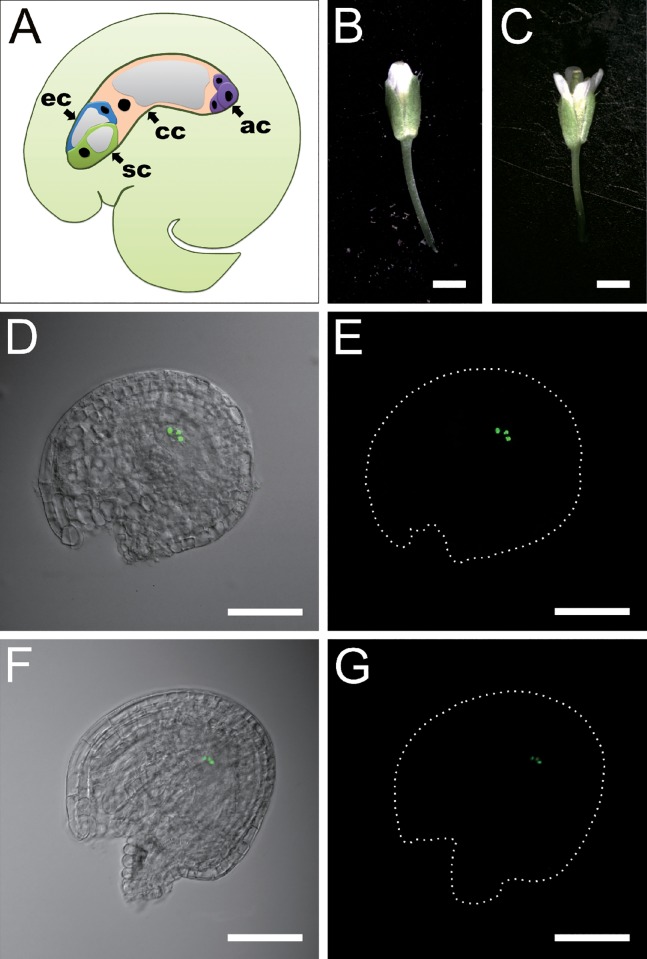
Test of antipodal cell maker line for antipodal cell isolation. **A**. Illustration of an ovule structure. sc: synergid cell; ec: egg cell; cc: central cell; ac: antipodal cell. **B**. A flower at the stage 12c. Bar = 1mm. **C**. A flower at the stage 14. Bar = 1mm. **D**. The ovules at flower stage 12c, a merged picture of GFP and DIC images. Bar = 50μm. **E**. Fluorescent image of the ovule at the stage 12c. Bar = 50μm. **F**. The ovules at flower stage 14 just after fertilization, a merged picture of fluorescence (*pDD13*::*H2B-GFP*) and DIC images. Bar = 50μm. **G**. Fluorescent image of the ovule at the stage 14. Bar = 50μm.

In *Arabidopsis thaliana*, the mature embryo sac consists of two synergid cells (sc) at the micropylar pole, one egg cell (ec), one central cell (cc) with one nucleus fused by two polar nuclei, and three antipodal cells (ac) located at the chalazal pole (Fig 1A). As the antipodal cells are very small in size in the embryo sac, it is difficult to locate them in a living ovule or in sections of an ovule. Therefore, we generated an antipodal cell marker line using the antipodal cell-specific promoter DD13[9] driving green fluorescent protein (GFP) and introduced it into wild-type *Arabidopsis* plants. We obtained stable marker lines, and among them, line K44-1 was selected for further analysis. We carefully observed the signals in the embryo sac throughout the whole process of ovule development. We found that at flower stage 12c, the GFP signal was strongest in mature embryo sacs (Fig 1B and 1D), and at stage 14, it was already weakening (Fig 1C and 1F). During these stages, GFP signals specifically appeared in the antipodal cells at the chalazal pole of ovules and were not found in other cell types. Thus, we chose line K44-1 and flowers at stage 12c as material for isolating antipodal cells.

### Procedure for Antipodal Cell Isolation and Observation of Isolated Antipodal Cells

**Fig 2:**
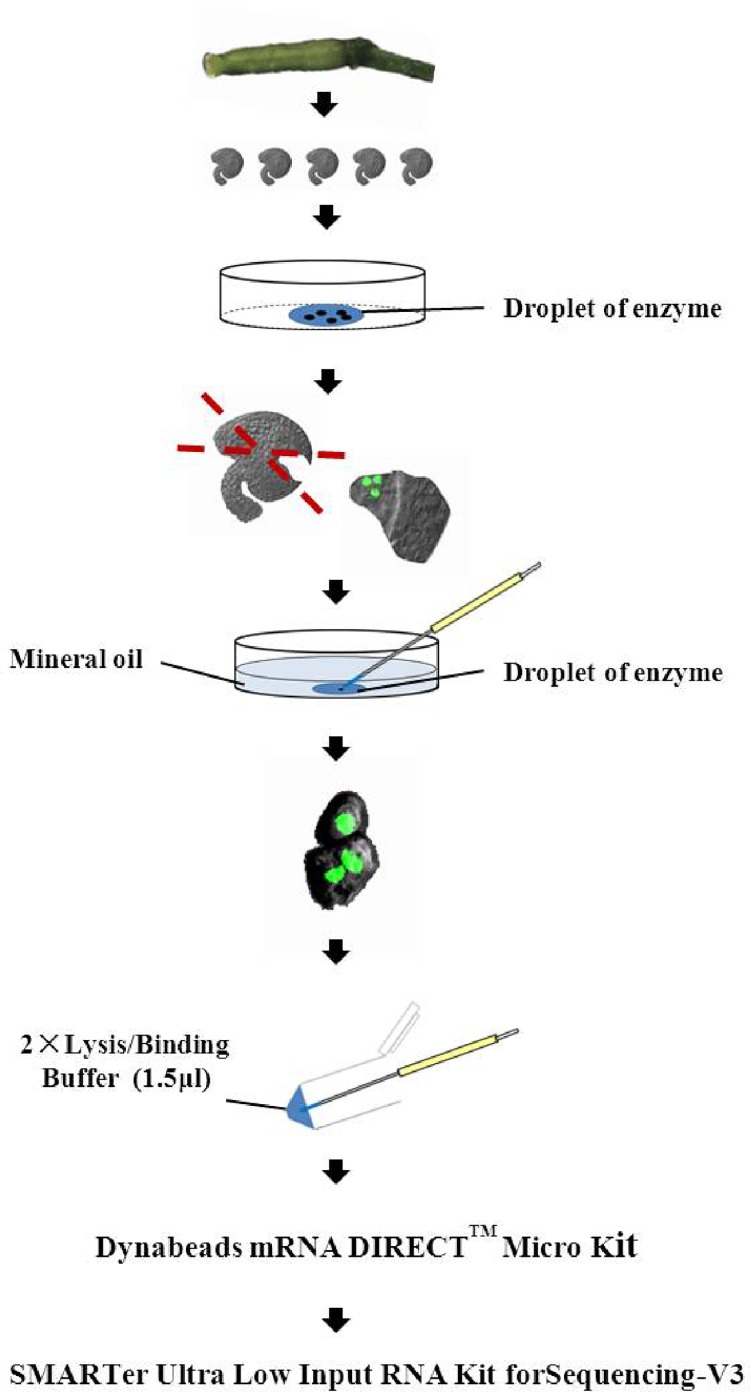
The procedure for the isolation of antipodal cells, mRNA extraction and cDNA amplification. Briefly, the ovules are dissected from flowers at the stage 12c and are further dissected by handmade razor under fluorescent microscope to obtain ovule fragments containing antipodal cells. The antipodal cells are further separated from other tissues by microneedles. The isolated antipodal cells are transferred into the 2x lysis buffer. Dynabeads are used for mRNA extraction and SMARTer Ultra Low Input RNA kit for Sequencing-v3 is used for cDNA amplification subsequently.

**Fig 3:**
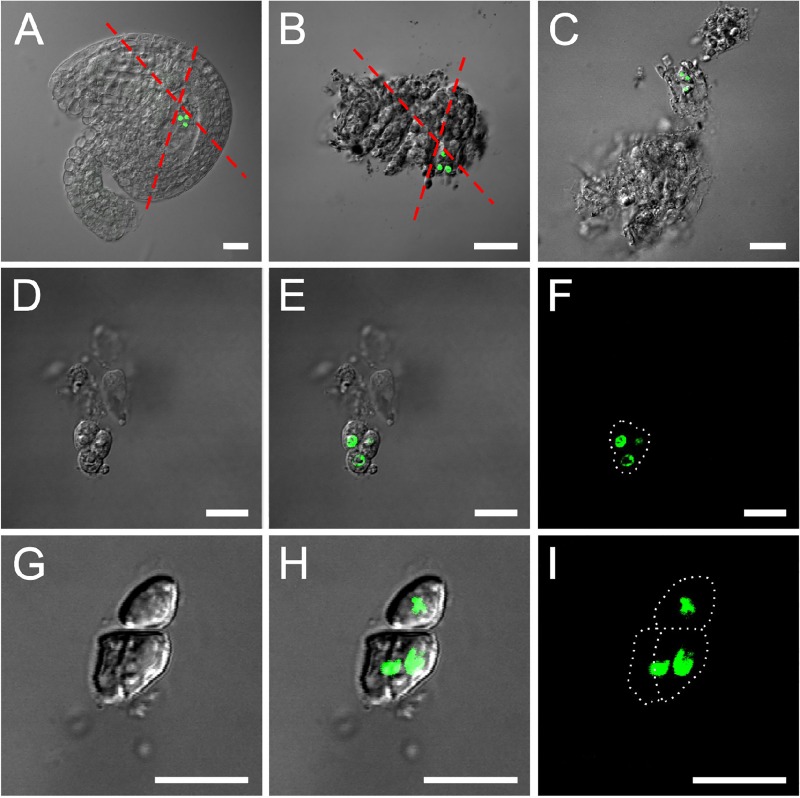
The technical illustration of antipodal cell isolation. **A**. The dissected ovule from flowers at the stage 12c. Red dash lines show the cutting direction. Bar = 20μm. **B.** The ovule fragment with sporophyte tissues. Red dash lines show the cutting direction. Bar = 20μm. **C**. The isolated ovule fragment with little sporophyte tissues from B. Bar = 20μm. **D**. The antipodal cells transferred into the new enzyme droplet, bright field image. Bar = 10μm. **E**. The antipodal cells transferred into the new enzyme droplet, the overlap of fluorescent (*pDD13*::*H2B-GFP*) and bright field images. Bar = 10μm. **F**. The antipodal cells transferred into the new enzyme droplet, a fluorescent image. Bar = 10μm. **G**. The isolated pure antipodal cells, bright field image. Bar = 10μm. **H**. The isolated pure antipodal cells, the overlap of fluorescent and bright field images. Bar = 10μm. **I**. The isolated pure antipodal cells, a fluorescent image. Bar = 10μm.

According to the characteristics of antipodal development and fluorescence of the marker line, we designed a reliable procedure for antipodal cell isolation after repeated tests (Fig 2). Ovules at stage 12c were first dissected and placed into a 100 μl enzyme droplet in a plastic dish (Φ 3.5 cm). The plastic culture dish was then placed under a fluorescent microscope to select ovules with clear fluorescent signals. The ovules with weak fluorescence were removed. To separate antipodal cells from the sticky inner layer of sporophyte tissue, a handmade razor blade was used to dissect the ovules. Each ovule was cut at least twice in proper directions to obtain the smallest ovule fragment containing antipodal cells (Fig 3A). It is important to make sure that one of the cutting lines is near the antipodal cells for the convenience of separating antipodal cells from surrounding tissues (Fig 3B). Since only antipodal cells showed GFP signals (Fig 3C), it was easy to recognize and locate them in cell mass. Antipodal cells stuck to other cells then were transferred into a new enzyme droplet in a culture dish (Φ 3.5 cm) and a handmade micropipette was used to pipette the cell cluster in and out gently for a few minutes to further separate the maternal tissues from the antipodal cells (Fig 3D–3F). In this whole process, the time that antipodal cells exposed in the enzyme buffer is about 10 minutes.

Once antipodal cells were isolated, they were washed twice in 10.5% (w/v) mannitol and transferred as soon as possible into 1.5 μl of 2x lysis buffer in a RNase-free PCR tube, and then stored at -80°C for subsequent experiments. The buffer volume of each transfer should be less than 1.5 μl to avoid over-dilution of templates. The total time for isolating a triple of antipodal cells is controlled in 15~30 minutes.

Using the method described above, we isolated pure antipodal cells without any contamination. Under our conditions, the length of the isolated antipodal cells were about 5 μm, much smaller than the length of egg cells (~30 μm) [14]. Three antipodal cells were always bound together, even in the enzyme buffer, which may be due to the interconnection via plasmodesmata [15] (Fig 2G–2I).

### Assessment of Antipodal Cell cDNA Quality

**Fig 4:**
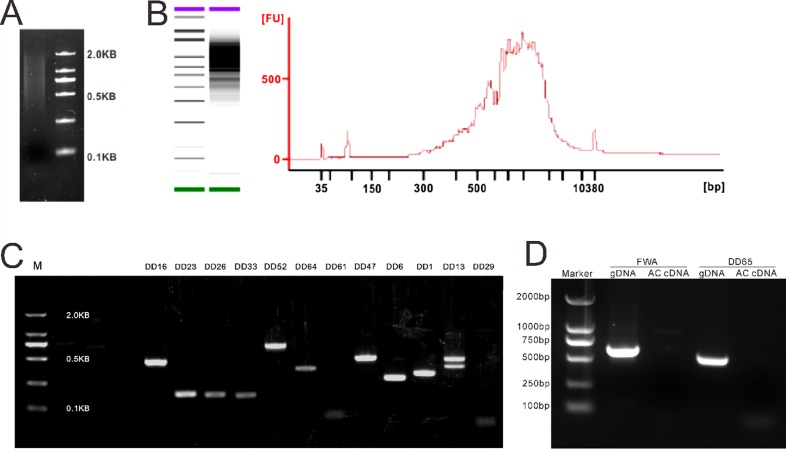
Assessment of antipodal cells’ cDNA quality. **A**. The gel electrophoresis analysis of antipodal cell cDNA. **B**. Alignment 2100 Bioanalyzer analysis of antipodal cell cDNA. Ladders from the bottom to the top are 35 (green), 50, 100, 150, 200, 300, 400, 500, 600,700, 1000, 2000, 3000 and 7000bp (purple) respectively. **C**. The verification of antipodal cell-specific gene expression by semiquantitative RT-PCR. **D**. The central cell specific maker FWA and DD45 were used as control to test possible contamination.

When a sufficient amount of antipodal cells were isolated (we used 108 antipodal cells), the Dynabead mRNA DIRECT Micro Kit (Invitrogen) was used for mRNA extraction, and the SMARTer® Ultra™ Low Input RNA Kit for sequencing-v3 (Clonetech) was used for mRNA reverse transcription and cDNA amplification to obtain sufficient cDNA for the following experiments. Agarose gel electrophoresis (Fig 4A) and Alignment 2100 Bioanalyzer (Fig 4B) showed that the cDNA of antipodal cells was mainly distributed from 500 bp to 2,000 bp. The concentration of cDNA reached 40.4 ng/μl, indicating that the mRNA of antipodal cells was of good quality and could be used for PCR screening and transcriptome analysis.

To further evaluate the cDNA quality of isolated antipodal cells, we detected 12 previously reported antipodal cell-specific genes, namely *DD1*, *DD6*, *DD13*, *DD16*, *DD23*, *DD26*, *DD29*, *DD33*, *DD47*, *DD52*, *DD61*, and *DD64* [9]. Our results showed that 10 of the 12 genes could be amplified from our cDNA sample by gene specific primers (Fig 4C; Table 1), indicating that that antipodal cells and RNAs isolated using our method could be used for gene expression analysis and antipodal cell-specific gene screening. Among them, *DD29* and *DD61* were not amplified from our cDNA sample. These genes may not be expressed during this stage, or isolated antipodal cells may have a low RNA abundance of these two genes.

**Table 1:** Primers used in our experiments.

Genes	Primers(5’-3’)
DD13 Pro	FW:NNNNGGTACCATCTTTTTGATAAATGAGAGTAC
DD13 Pro	RE:NNNNCTCGAGTCTCAAAATCTGCATATATCTTTTTAATGAC
DD1	FW: GCACTCCCTATGAAGGTGGC
DD1	RE: TTCTCGAGCTCTCTGGTCAA
DD6	FW: CTTGCTGCACAGCAGAACAC
DD6	RE:GCAGCACCATTGAAATCGCA
DD13	FW:TGTCGTTAGGCAAGGCATCA
DD13	RE:TGAGCTTTTGGATCGAGCGT
DD16	FW:TGGCACATTCCTCTGTTGCT
DD16	RE:GCATCCCGAGACAGATACCG
DD23	FW:AAAGCCGGTGGTTCTCATGT
DD23	RE:TGGATCGTGTTTCGGGTCTG
DD26	FW:CTCCTAGACGCCCCCTTACT
DD26	RE:CCTCCTATTCCGTTTGTGGGT
DD29	FW:GACCGTCCAAAGTGCAATCG
DD29	RE:CCCAACAACGTTTTCCCGAC
DD33	FW:GATCCGATGTGTCCTGGAGT
DD33	RE:TGGTGGAACATCAACAGGTCC
DD47	FW:TAACGAATTCCGGCGTCACA
DD47	RE:CCCACATGTTCGAATCCCCA
DD52	FW:TGACACGTGTTACGGTCCTG
DD52	RE:ACTCGCTCAAACCCTCCTTG
DD61	FW:GCATACCGGCATTTCCCAAC
DD61	RE:ACAGAGCGAAGTTCTGACCG
DD64	FW: GCCGGAGAAAACGAAACGAC
DD64	RE:TGCCCCTTTTTCCCCATTCA
FWA	FW:TGGTGGAACCAAAAGTGGCT
FWA	RE: ATGCACTCAGCACAATCCCA
DD65	FW: TGGGCTAAAAACACAGGCCA
DD65	RE: GTGTGTTCGTTGGTTTCCCG

As central cell is adjacent to the antipodal cells and occupies the major volume of an embryo sac, it is considerable that the antipodal cells cDNA might be contaminated by the central cells. For this reason, two central cell marker genes, FWA[16] and DD65[14], were used to test the possibility of central cell contamination in the isolated antipodal cells by Polymerase Chain Reaction (PCR) (Fig 4D). The genomic DNA (gDNA) was used as the control for primer’s validity. The results showed that both FWA and DD65 cannot be amplified when antipodal cell cDNA (AC cDNA) was used as the template, indicating that the antipodal cell cDNA is not contaminated by central cells.

### *In Vitro* Development of the Isolated Antipodal Cells

**Fig 5:**
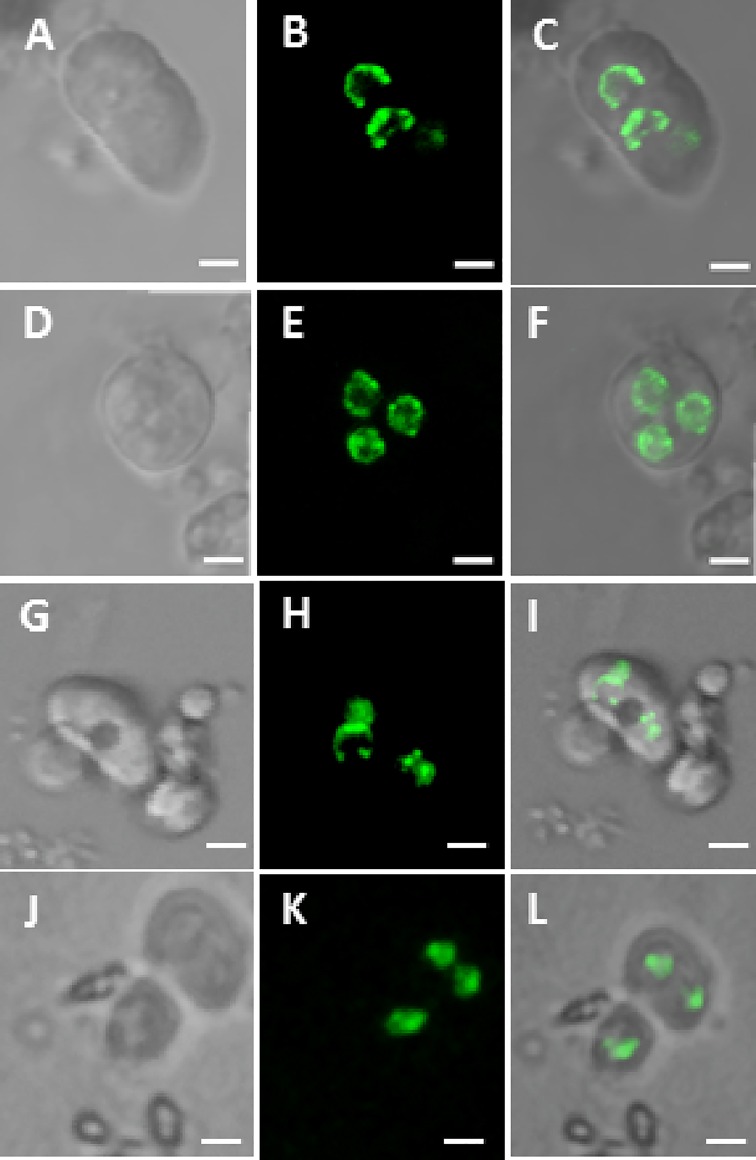
*In vitro* culture of isolated antipodal cells. A-B. Confocal images of just isolated antipodal cells. D-F. Confocal images of antipodal cells after 3–5 hours’ culture. Notice the dotted fluorescence. G-I. Fluorescent microscopic images of antipodal cells after one week culture. J-L. Fluorescent microscopic images of antipodal cells in the culture chamber after one week culture. Notice the condensed fluorescence of nuclei. Bar = 5μm.

By the means of microculture, we tried to observe the *in vitro* development of the isolated antipodal cells. As soon as the cells were released, they were put in a microchamber (a Millicell) containing culture medium according to the previously established method [17]. Under this culture condition, the major development events of these isolated antipodal cells could be observed. These cultured antipodal cells could keep their viability more than one week, while their counterparts *in vivo* are usually degenerated via programed cell death (PCD) within three days after embryo sac maturation. This suggests that the PCD process was likely interrupted due to cell culture and thus the antipodal cell could keep intact and viable after one-week culture, although no cell division was observed yet. Accordingly, the cultured cells underwent some cytological changes. Just isolated antipodal cells usually showed loosely distributed DNA fluorescence and micronuclei, a typically character of PCD (Fig 5A–5C), after few hours’ culture, the fluorescence was getting denser and evener distributed (Fig 5D–5F). After one week couture, some of the cells remained this status (Fig 5G–5I), but some other cells showed dramatic changes, the micronuclei were almost disappeared and the fluorescence became concentrated and looked like normal nuclei (Fig 5J–5L). This indicates the *in vitro* developmental potential and cell fate transition of the isolated antipodal cells in culture condition. Obviously, to trace the cell fate transition and confirm their cell totipotency, much more careful and hard work should be done in the future.

## Discussion

Methods for isolating female gametes and accessory cells have been developed in *Zea mays* [18], *Triticum aestivum* [19], *Oryza sativa* [20], and tobacco [21]. In maize, egg cells are isolated by enzymatic treatment followed by mechanical isolation [18]. In wheat, a method without enzymatic treatment was developed to isolate egg cells [19]. In rice, both enzyme-treated manipulation methods and direct isolation without enzymatic treatment were established to isolate both egg cells and central cells [20]. In tobacco, enzymatic treatment combined with osmotic pressure is used for isolating egg cells and synergid [21]. However, in *Arabidopsis thaliana* (a model plant), there is no reported method to isolate synergid cells, egg cells, central cells, or antipodal cells. Among these cell types, antipodal cells are the most difficult cell type to isolate due to their small size and specific location at the chalazal pole of the ovule. At this time, the only possible method to stably obtain these cells is laser-assisted micro dissection (LAM), which has been used to isolate synergid cells, egg cells, and central cells, but not antipodal cells [22]. Taking into the consideration the small size and location of antipodal cells, application of the LAM method for antipodal cell isolation is very difficult.

Considering the structure of the mature embryo sac in Arabidopsis and based on experience in maize, wheat, tobacco and rice, we developed a reliable method to isolate antipodal cells, the smallest cells in the embryo sac. Using this method, we were able to isolate living antipodal cells, and the isolated cells could be used for specific experiments such as live imaging and *in vitro* culture. As described above, this method can isolate pure antipodal cells without contamination from sporophyte tissues. It is important to identify genes specifically expressed in antipodal cells, and this method can be used for transcriptome analyses.

To apply this method, some manipulation details should be addressed. First, as the isolation process can cause stress to the cells, which may induce stress responsive gene expression, the isolation process should be minimized. Second, to maintain the quality of the isolated cells, we collected each group of antipodal cells (three antipodal cells) immediately after isolation and did not accumulate isolated cells in a Petri dish; that is, we transferred them into lysis buffer for storage as soon as possible. Third, it is important to control the volume of liquid when transferring cells into the lysis buffer. As the cell number increased by several transfers, the volume of the buffer also increased. The total volume of the lysis buffer should be limited to 3 μl to avoid dilution of the templates and to ensure successful amplification, since there is only 1.5 μl of lysis buffer (2X) in each tube for storage.

## Conclusions

We established a method for efficient antipodal cell isolation in *Arabidopsis thaliana*. This technique allows for the collection of pure antipodal cells and can be used for screening antipodal cell-specific genes and transcriptome analysis. This is the first method for isolating antipodal cells and can be also used for isolating other cell types in the embryo sac, such as egg cells.
